# Bioinformatics‐based discovery of intervertebral disc degeneration biomarkers and immune‐inflammatory infiltrates

**DOI:** 10.1002/jsp2.1311

**Published:** 2023-12-22

**Authors:** Chao Song, Daqian Zhou, Kang Cheng, Fei Liu, Weiye Cai, Yongliang Mei, Jingwen Chen, Chenyi Huang, Zongchao Liu

**Affiliations:** ^1^ Department of Orthopedics and Traumatology (Trauma and Bone‐setting), Laboratory of Integrated Chinese and Western Medicine for Orthopedic and Traumatic Diseases Prevention and Treatment The Affiliated Traditional Chinese Medicine Hospital of Southwest Medical University Luzhou Sichuan China; ^2^ RuiKang Hospital affiliated to Guangxi University of Chinese Medicine Nanning Guangxi China; ^3^ Luzhou Longmatan District People's Hospital Luzhou Sichuan China

**Keywords:** biomarkers, cellular senescence, differentially expressed genes, GEO, immune inflammatory, intervertebral disc degeneration

## Abstract

**Background:**

Intervertebral disc degeneration (IVDD) is a common chronic disease in orthopedics, and its molecular mechanisms are still not well explained.

**Aim:**

This study's objective was to bioinformatics‐based discovery of IVDD biomarkers and immune‐inflammatory infiltrates.

**Materials and Methods:**

The IVDD illness gene collection was gathered from GeneCards, DisGeNet, and gene expression profiles were chosen from the extensive Gene Expression Omnibus database (GSE124272, GSE150408, and GSE153761). The STRING database was used to create a network of protein–protein interactions, while the Kyoto Encyclopedia of Genes and Genomes (KEGG) and Gene Ontology (GO) databases were used for functional enrichment analysis. Using hub genes, the immune cell infiltration between IVDD patient samples and control tissues was examined. Finally, quantitative polymerase chain reaction and Western blot experiments were used to verify the expression of hub genes.

**Results:**

A total of 27 differentially expressed hub genes were identified by bioinformatics. According to GO and KEGG analyses, hub genes were prominent in immunological responses, chemokine‐mediated signaling pathways, and inflammatory responses, with the key signaling pathways engaged in cellular senescence, apoptosis, Th1 and Th2 cell differentiation, and Th17 cell differentiation. Immune cell infiltration research revealed that T cells, lymphocytes, B cells, and NK cells were decreased in IVDD patients while monocytes, neutrophils, and CD8 T cells were increased. The expression levels of the senescence hub genes SP1, VEGFA, IL‐6, and the apoptosis key gene CASP3 were considerably greater in the IVDD model group than in the control group, according to in vitro validation.

**Conclusion:**

In conclusion, the cellular senescence signaling pathway, the apoptosis signaling pathway, and associated hub genes play significant roles in the development and progression of IVDD, this finding may help direct future research on the senescence signaling route in IVDD.

## Graphical abstract

Intervertebral disc degeneration (IVDD) is a common chronic disease in orthopedics, and its molecular mechanisms are still not well explained. The pathophysiology of IVDD is primarily related to aberrant expression of signaling pathways such as cellular senescence, apoptosis, and Th17. Four hub genes—SP1, CASP3, VEGFA, and IL‐6—can be used to diagnose IVDD, which is advantageous for its prevention and therapy.

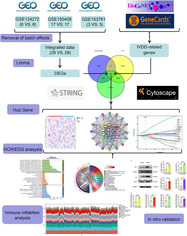

## 1 INTRODUCTION

Intervertebral disc degeneration (IVDD) is a musculoskeletal degenerative condition associated with aging that lowers people's quality of life and places a greater financial burden on society.^1^ The pathogenesis of IVDD is now thought to be primarily caused by cellular senescence (CS) or death of nucleus pulposus cells (NPCs), an imbalance of extracellular matrix (ECM) formation and degradation, and inflammation, oxidative stress, but the precise pathophysiology is not entirely understood.^2^, ^3^ Therefore, it is necessary to explore the pathogenesis of IVDD. Conservative therapy and surgical treatment comprise the majority of the current clinical care of IVDD.^4^, ^5^ Conservative treatment mainly includes oral pain medication, functional exercises, acupuncture, and massage, as well as promising treatments such as gene therapy, molecular therapy, and biologics that are being investigated.^4^, ^5^ Surgical treatment options include spinal decompression, lumbar fusion, and discectomy. However, none of these treatments can reverse the biomechanical changes in the entire spine caused by disc degeneration.^6^, ^7^ As a result, it is critical to identify patients who are at high risk for IVDD early on to help clinicians decide which therapeutic choices to use.

CS is an important cause of disc degeneration, which is classically defined as irreversible cell cycle arrest caused by telomere shortening or various external stimuli.^8^, ^9^, ^10^ Senescent cells no longer have the capacity to divide, but they inappropriately release inflammatory cytokines, matrix‐degrading proteases, growth factors, and chemokines that cause immunological dysregulation and worsen the clinical symptoms of IVDD.^11^ This proinflammatory phenotype of senescent cells is defined as the senescence‐associated secretory phenotype (SASP).^12^ Senescent cells release SASP‐related proteins such as ECM proteases, proinflammatory factors (IL‐6, TNF‐α, IL‐7, IL‐8), cytokines, and other bioactive compounds that can increase the production of ROS, DNA damage, and mitochondrial dysfunction, which can result in IVDD.^11^ Senescent cells, on the other hand, can contribute to the breakdown of the disc's ECM and inflammation by secreting metabolic factors, luring inflammatory cells, inducing senescence in nearby cells through paracrine secretion, encouraging peri‐disc cell senescence, and promoting the infiltration and differentiation of immune cells. This worsens the inflammatory immune response in the microenvironment of the degenerated disc.^13^, ^14^ Therefore, a potential therapeutic approach to treat IVDD may involve reducing immune inflammation and disc cell senescence.

The purpose of our article is to discover the potential mechanisms of inflammation, apoptosis, and senescence‐related genes in IVDD through bioinformatics approaches, and to provide a theoretical basis for subsequent studies by verifying the existence of related genes through preliminary experiments. Based on bioinformatic analysis, we evaluated genes linked to senescence signaling pathways, apoptotic signaling pathways, and immunity by carefully examining the GSE124272, GSE150408, and GSE153761 datasets in the Gene Expression Omnibus (GEO) database, as well as collecting IVDD disease datasets, making a significant contribution to our study.^15^, ^16^, ^17^ To forecast the involvement of senescence signaling routes, apoptotic signaling pathways, and immune signaling pathways in IVDD, we first acquired a list of 27 key genes for IVDD. Based on this, we did an enrichment analysis of pivotal genes. In addition, we discovered immune cell infiltration of the hub genes, which is consistent with differential expression of associated genes during the pathogenesis of IVDD, demonstrating the viability of the model we created for IVDD diagnosis. In addition, it will give a solid theoretical framework for future research on aging and immunological inflammation in IVDD.

## 2 MATERIALS AND METHODS

### 2.1 Microarray data source and differential gene analysis

Datasets GSE124272, GSE150408, and GSE153761 were downloaded from the GEO database.^15^, ^16^, ^17^, ^18^ Microarray data for GSE150408 comprises 17 IVDD samples, 17 normal control samples, and 25 treatment samples, whereas microarray data for GSE124272 contains 8 IVDD samples and 8 normal control samples, GSE153761 contains 3 IVDD samples, and 3 normal control samples. The GSE153761 dataset was sequenced on GPL22120, whereas the GSE124272 and GSE150408 datasets were sequenced on GPL21185. GSE150408 data were from 17 patients with sciatica confirmed by magnetic resonance imaging and 17 healthy volunteers without clinical evidence of low back pain or sciatica. The specimen collected was a blood specimen from the participant's left median elbow vein. GSE124272 data are from eight patients with lumbar disc prolapse confirmed by magnetic resonance imaging, and eight volunteers without clinical evidence of low back pain or sciatica. The specimens collected were left median elbow vein blood specimens from the participants. GSE153761 data were obtained from three healthy subjects from patients with cervical spine fractures who underwent anterior cervical discectomy and fusion, and three samples of cartilage endplates from cervical disc degeneration. The above specimens did not elucidate the grade of degeneration, the gender of the volunteers, or the age of the patients (see Supporting Information Material for specific information, original data). Both datasets used humans as their sources and underwent Gene Symbol modification. Sangerbox 3.0 was used to de‐batch the aforementioned three datasets to construct the consolidated GEO dataset.^19^

Investigated the effects of pertinent gene expression levels on IVDD using differential gene analysis utilizing the “limma” function of Sangerbox 3.0 to find differential genes between IVDD samples and control samples in the integrated dataset.^19^ The absolute value of log2 fold change |log2FC| >1.2 and *p* < 0.05 were used as the criterion for differentially expressed genes (DEGs), suggesting upregulation of DEGs. *p* < 0.05 and |log2FC| <1.2 both showed downregulation of DEGs. Volcano and heat maps are used to show the results of differential gene expression.

### 2.2 Acquiring the IVDD disease dataset

GeneCards (https://www.genecards.org/) and DisGeNet (https://www.disgenet.org/), databases were used to get the target proteins of the disease, using the keyword “Intervertebral disc degeneration,” to further research the causes and consequences of IVDD.^20^, ^21^ The search results from these databases were combined and de‐duplicated to obtain the disease gene sets for IVDD.

### 2.3 Identification of IVDD biomarkers

We intersected the Gene Symbol from the IVDD illness gene dataset (which refers to disease data genes obtained from GeneCards, DisGeNet in Methods 2) with the GEO integrated dataset to obtain the intersecting gene set to precisely identify critical biomarkers that contribute to the IVDD process. The critical genes for the IVDD process are found in this intersecting gene group. The majority of biological activities in live cells are governed by protein interactions, which are crucial for comprehending cellular physiology in both healthy and pathological conditions. Using the string database (http://string-db.org/) and a confidence value >0.4, PPI network analysis was carried out in this study on the “*Homo sapiens*”‐restricted set of intersecting genes. PPI network created using Cytoscape (version 3.9.1) software.^22^ In addition, the CytoHubba algorithm (Maximum Neighborhood Component[DMNC], Maximum Neighborhood Component [MNC], Edge Percolated Component [EPC], Closeness, Betweenness, Clustering Coefficient, EcCentricity, Radiality, Stress, Bottle Neck), a plug‐in to Cytoscape software, was used to obtain key gene set.^23^ The critical gene list was screened using the CytoHubba algorithm to identify hub genes with substantial roles, helping to further narrow down the genes involved in the IVDD process. The hub genes were added to the GEO integrated dataset to extract the differential gene expression between IVDD samples with normal samples, and the findings of the differential gene expression were displayed using differential volcano plots and box‐line plots.

### 2.4 Construction of trait genes and survival analysis

The model was trained to predict the likelihood of IVDD using the least absolute shrinkage and selection operator (LASSO) technique. The LASSO technique was used to analyze candidate differentially expressed hub genes and identify hallmark genes linked to IVDD.^24^ Forest models were used to predict the likelihood of different pivotal genes for IVDD, and ROC curves were used to assess the efficacy of pivotal genes overall.^25^

### 2.5 Enrichment analysis

Used the Sangerbox 3.0 BioCloud platform to import the hub gene set, then restricted the species to “*H. sapiens*” in the Tools Centre and chose Enrichment Analysis. To submit, enter the hub gene's gene symbol after setting the common parameters. The hub genes' final Gene Ontology (GO) enrichment analysis and Kyoto Encyclopedia of Genes and Genomes (KEGG) database pathway analysis were conducted, and the final results of the analysis are presented in different graphs.^26^, ^27^

### 2.6 Identification of immune‐infiltrating cells in disease

The immune microenvironment was generally composed of immune cells, inflammatory cells, fibroblasts, mesenchymal cells, different cytokines, and chemokines.^28^ Immune cell infiltration analysis is an important guide in predicting the course of disease and response to treatment. The single‐sample Gene Set Enrichment Analysis algorithm is an extension of the GSEA method for quantifying the abundance of 28 immune cell types in individuals with different immune patterns. The CIBERSORT algorithm can perform linear support vector regression to deconvolute gene expression profiles and use RNA sequencing data to estimate the number of immune cells in a sample.^29^ The immune cell types of patients with various immunological patterns were determined using the Sangerbox 3.0 software's CIBERSORT algorithm, and their immune cell composition was displayed using stacked charts. Finally, using the Wilcoxon rank sum test, changes in immune cell proportions were evaluated.

### 2.7 Western blot, qRT‐PCR validation of hub genes

To further clarify the differences in cell senescence and apoptosis hub genes SP1, CASP3, VEGFA, and IL‐6 in IVDD, an IVDD model was constructed using lipopolysaccharide‐induced NPCs, which are the key cells in the intervertebral disc structure, senescence and inflammation of NPCs play an important role in IVDD and NPCs are easy to obtain and easy to experiment, therefore, we chose NPCs for the validation, and protein and RNA differences between the IVDD model group and the normal group were examined by Western blot (WB) and qRT‐PCR.

#### 2.7.1 Experimental items

Human NPCs‐immortalized (HUM‐iCELL‐s012) were purchased from the cell bank of the Chinese Academy of Sciences, and derived from intervertebral disc tissue are lentivirally transfected to carry the SV40 gene. LPS was purchased from Aladdin Company. HPLC grade (≥94%); PC‐1iCell Primary Chondrocyte Cell Culture System was purchased from HyClone Inc.; fetal bovine serum (FBS) was purchased from Gibco Inc. SP1, CASP3, IL‐6, VEGF antibody and GAPDH antibody were purchased from Biobay Bio. CCK‐8 kit (cell proliferation and toxicity detection kit) was purchased from Beijing Solabio Technology Co.

#### 2.7.2 LPS solution preparation

LPS (100 mmol/L) stock solution was prepared by dissolving 10 mg of LPS solution in 438 μL of dimethyl sulfoxide and packaged and stored in a −20°C refrigerator, it was diluted to the following concentrations using the PC‐1iCell Primary Chondrocyte Cell Culture System (containing 10% FBS): 0.01, 0.1, 1, 10, and 100 μmol /L.

#### 2.7.3 Cell culture

HUM‐iCELL‐s012, so that the PC‐1iCell Primary Chondrocyte Culture System (PriMed‐iCell‐020) was cultured at 37°C in a 5% carbon dioxide incubator, and all the experiments were carried out when the cells were grown to more than 80%.

#### 2.7.4 The effect of drugs on the proliferative function of cells was tested by CCK‐8 assay

NPCs were grown to 80% fusion and digested with digest containing 0.25% trypsin to make cell suspension. Cells were seeded in 96‐well plates 3000 cells/well and cultured in a 5% CO_2_ incubator at 37°C. After 24 h of cell attachment, 200 μL of medium containing different concentrations of LPS (0.01, 0.1, 0.1, 1, 10, and 100 μmol/L) was intervened, and cells were cultured for 24 and 48 h. The cell proliferation function was determined by adding 10 μL of CCK‐8 solution to each well. Ten microliters of CCK‐8 solution were added to each well and incubated in an incubator for 2 h. The OD value at 450 nm was read with a multifunctional enzyme marker.

#### 2.7.5 WB for protein expression

The optimal time and concentration of LPS intervention were selected by CCK‐8 as the model group, and normal NPCs were used as the control group. The approximate WB procedure was as follows: the NPCs tissue from each group was rinsed twice with cold phosphate‐buffered saline and then homogenously lysed with 1 mL of ice lysis buffer. The supernatant was collected, incubated on ice for 20 min, and then centrifuged at 12 000 rpm for 20 min at 4°C. The total protein content was determined using a bichondroitin acid protein assay kit. The same amount of protein was extracted from each sample using sodium dodecyl sulfate–polyacrylamide gel electrophoresis; the separated proteins were then transferred onto polyvinylidene difluoride (PVDF) membranes. After being sealed in 5% skimmed milk for 2 h at room temperature, the membrane was incubated with the primary antibody overnight at 4°C. The PVDF membranes were then treated with a secondary antibody for 2 h at room temperature. Select β‐actin as the reference protein. The images were densitometrically analyzed using ImageJ software version 1.8.

#### 2.7.6 Real‐time fluorescence quantitative polymerase chain reaction for RNA expression

Similarly, the optimal time and concentration of LPS intervention were selected by CCK‐8 as the model group, and normal NPCs were used as the control group. qRT‐PCR procedure: Total RNA was extracted from the control and model group using the Total RNA Extraction Kit. All RNA was then reverse transcribed into cDNA using the iScriptcDNA Synthesis Kit, relative mRNA levels were calculated. GAPDH was normalized using the 2^−ΔΔ*C*t^ technique and analyzed using the Bio‐RadCFX96 device for real‐time fluorescence quantitative polymerase chain reaction (qPCR) analysis. Genes encoding SP1, CASP3, VEGFA, and IL‐6 were obtained for the final list of primer sequences.

### 2.8 Statistical analysis

GraphPad Prism 9.0 software was used for statistical analysis and graphing. Data are presented as histograms of means ± standard error of the mean values for data from three or more independent experiments. If the samples fell under the normal distribution, the *t*‐test was used to compare the two groups, and the nonparametric test was used if they did not. One‐way analysis of variance was used to compare samples from different groups. Tukey's method test was used to compare any two groups, with *p* < 0.05 denoting a statistically significant difference and *p* < 0.01 denoting a statistically significant difference.

## 3 RESULTS

### 3.1 Results of differential gene analysis based on different subgroups

**FIGURE 1:**
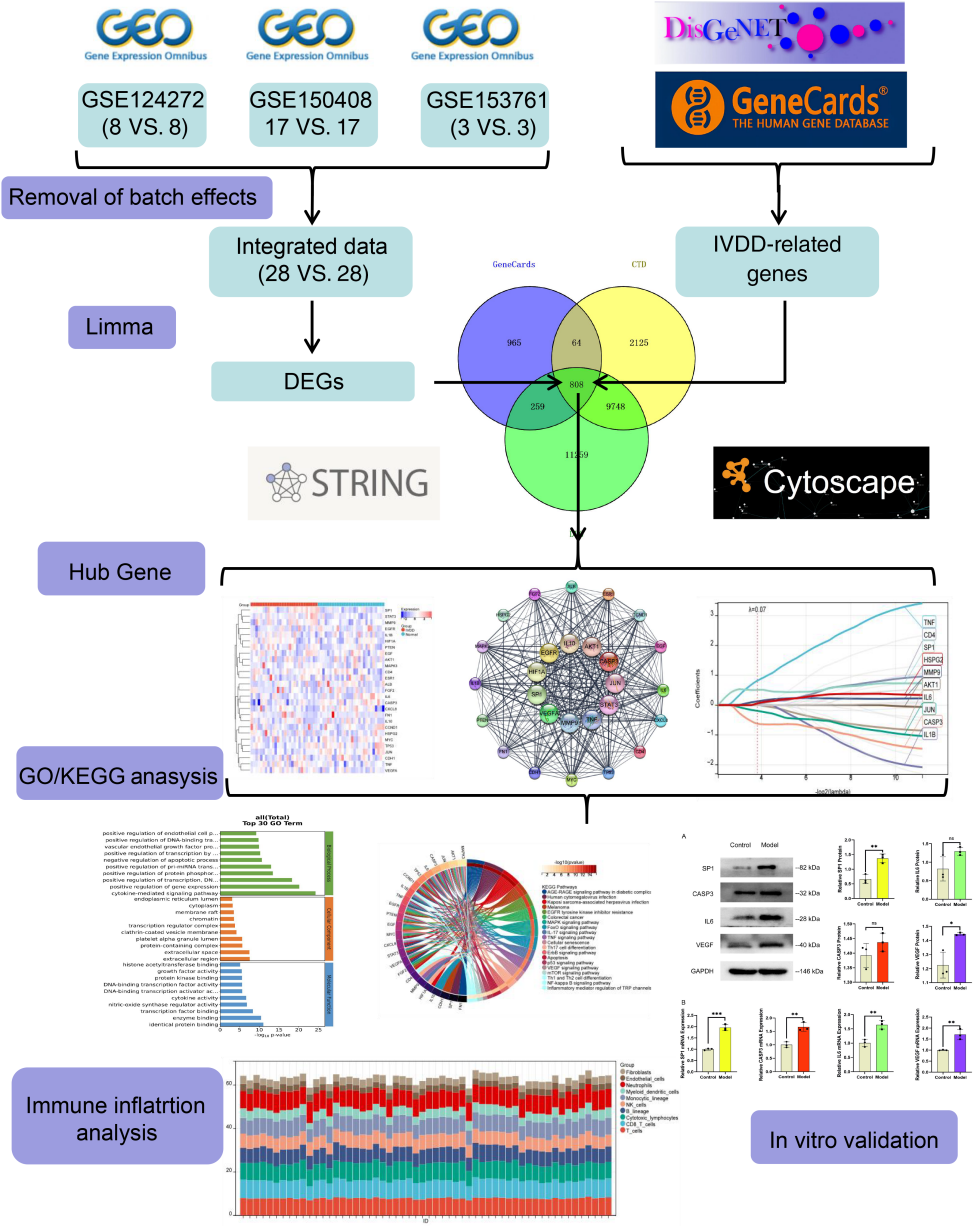
This diagram shows the flow structure of the entire article.

**FIGURE 2:**
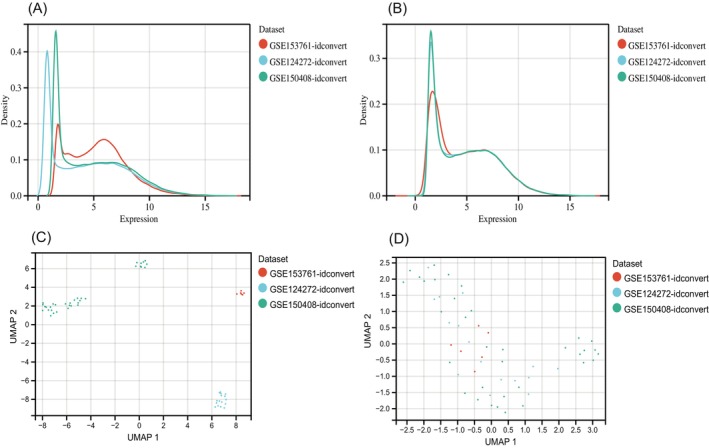
(A) From the density plot, the distribution of samples in each dataset varied greatly. (B) After the batch effect was removed, with similar means and variances. (C) From the UMAP plot, the samples in each dataset clustered together. (D) After removing the batch effect, a good removal of the batch effect.

**FIGURE 3:**
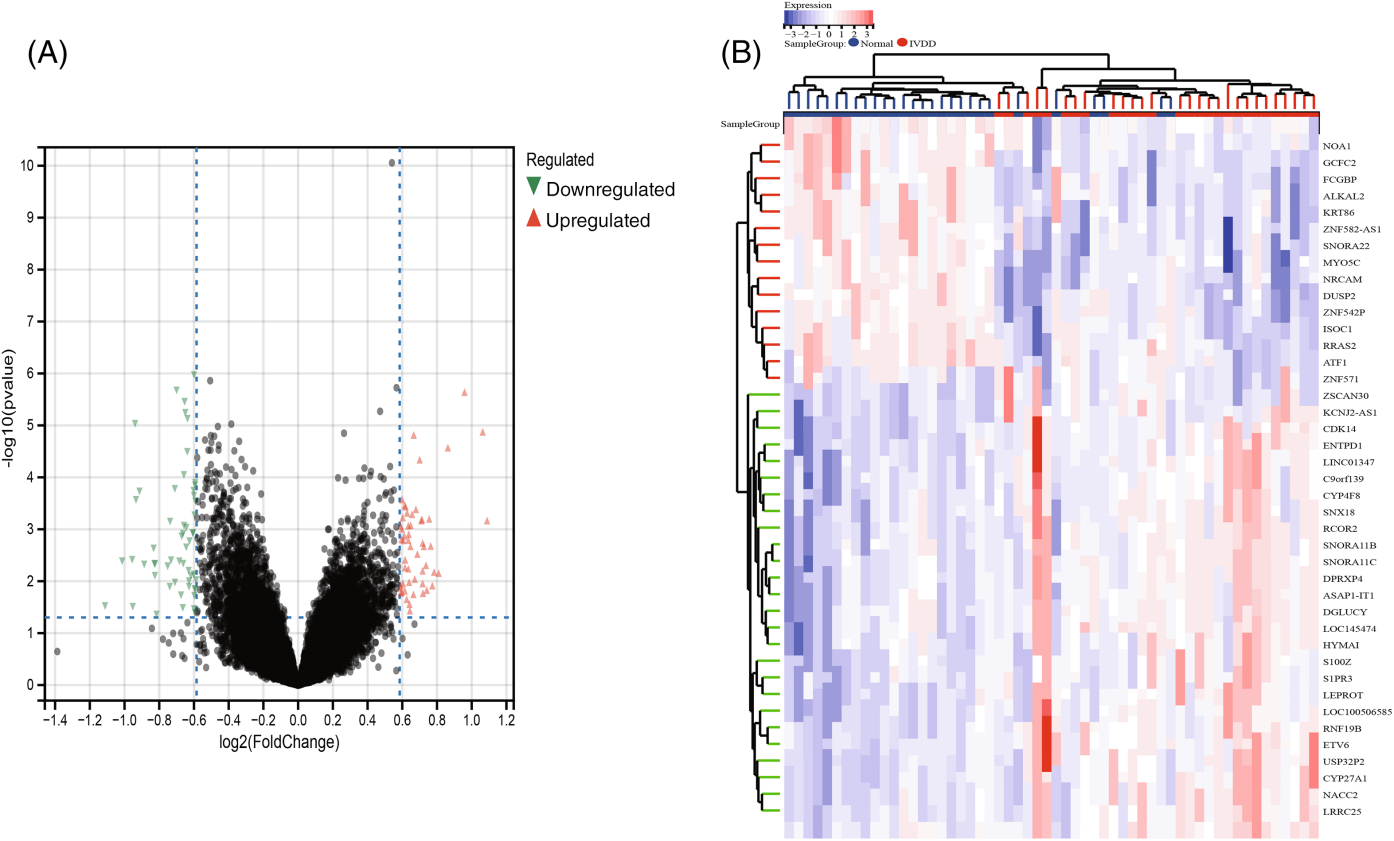
Limma analysis. (A). The differential volcano plot shows differential gene expression in general, with red triangles indicating upregulated genes, green triangles indicating. (B) The difference heat map clearly shows the genes with significant differences between groups, with red indicating upregulation and blue indicating downregulation.

Figure 1 depicts the bioinformatics analysis flow for this project. The GEO dataset, which included 28 IVDD samples and 28 normal control samples, was subjected to a batch treatment removal process, and 25 treatment samples were left out (Table 1; Figure 2). Out of 22 074 genes, a differential analysis of IVDD samples and control samples identified 151 DEGs, of which 87 had their expression upregulated and 64 had it downregulated (Figure 3A,B).

**TABLE 1:** Descriptive statistics.

Data number	Platform information	IVDD group	Control group	Species
GSE124272	GPL21185	8	8	*Homo sapien*
GSE150408	GPL21185	17	17	*Homo sapien*
GSE153761	GPL22120	3	3	*Homo sapien*

### 3.2 Biomarkers of IVDD

**FIGURE 4:**
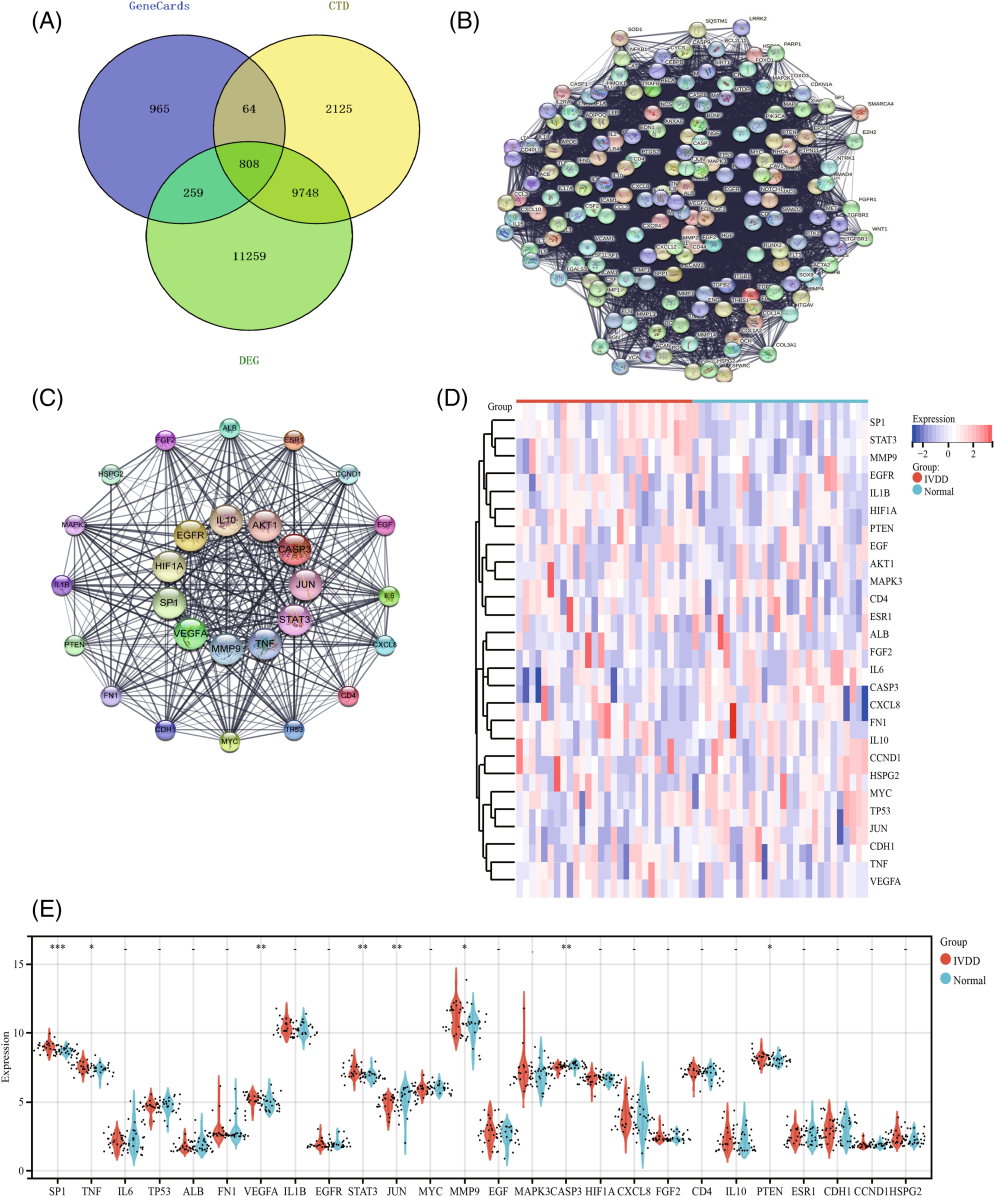
(A) Venn plot of 808 significant intersecting gene sets. (B) One hundred sixty‐two key gene sets were obtained after PPI screening. (C) Twenty‐seven hub gene sets were obtained after PPI screening. (D) Heat map of hub‐based differential expression between IVDD sample groups and normal controls. (E) The box‐line plot of pivotal‐based differential genes shows the statistics of differential expression (*n* = 28, ***p < 0.001; **p < 0.01; *p < 0.05, comparisons between the two of this group were made using a *t*‐test).

Eight hundred‐eight significant intersection gene sets were found after searching the GeneCards, DisGeNet illness database for the intersection of the disease targets with 22 074 matrix data (Table S1; Figure 4A). One hundred sixty‐two important gene sets and 27 hub genes were discovered by the PPI and Cytoscape screenings (Tables S2 and S3; Figure 4B,C). We examined the expression of 27 important genes after comparing them to the original differential expression data (Table S3). The differential gene box‐line plot and differential gene heat map both display the statistical situation of differential expression of pivotal genes in Figure 4D,E, respectively. The statistically significant hub genes are SP1, STAT3, MMP9, PTEN, CASP3, JUN, TNF, and VEGFA.

### 3.3 Results of characteristic genes and survival analyses

**FIGURE 5:**
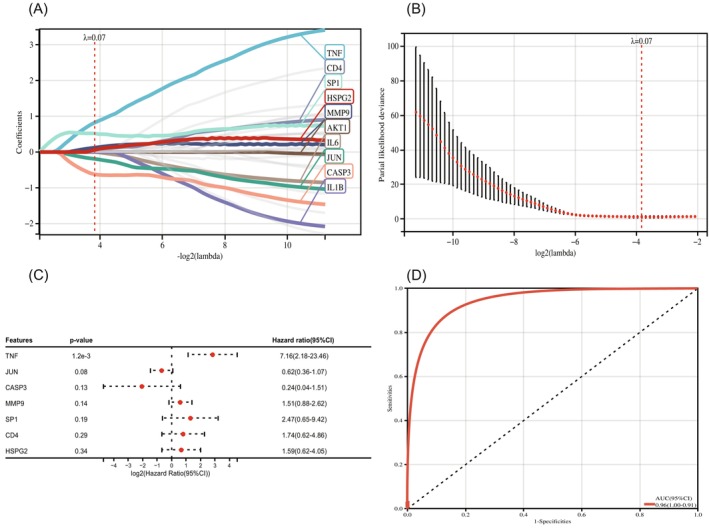
(A) Construction of the IVDD model using the LASSO algorithm hub genes for screening gene signatures, positive numbers indicate that upregulation of genes occurs in IVDD and negative numbers indicate that downregulation of genes occurs in IVDD. (B) Construction of the IVDD model using the LASSO algorithm hub genes for screening gene signature efficacy. (C) Forest plot of hub gene signatures of IVDD patients, positive numbers indicate that upregulation of a gene has occurred, and negative numbers indicate that downregulation of a gene has occurred. (D) Receiver operating characteristic (ROC) curve of predicted risk scores in IVDD diagnosis.

Using the LASSO algorithm, we obtained the signature genes associated with IVDD, the main genes involved were TNF, CD4, SP1, HSPG2, MMP9, AKT1, IL‐6, JUN, CASP3, IL‐1B (Figure 5A,B). Forest plots based on these signature genes showed that TNF, JUN, CASP3, MMP9, SP1, CD4, and HSPG2 were significant predictors of IVDD disease (Figure 5C). The final ROC curve showed better efficacy for these signature genes (Figure 5D).

### 3.4 Enrichment analysis

**FIGURE 6:**
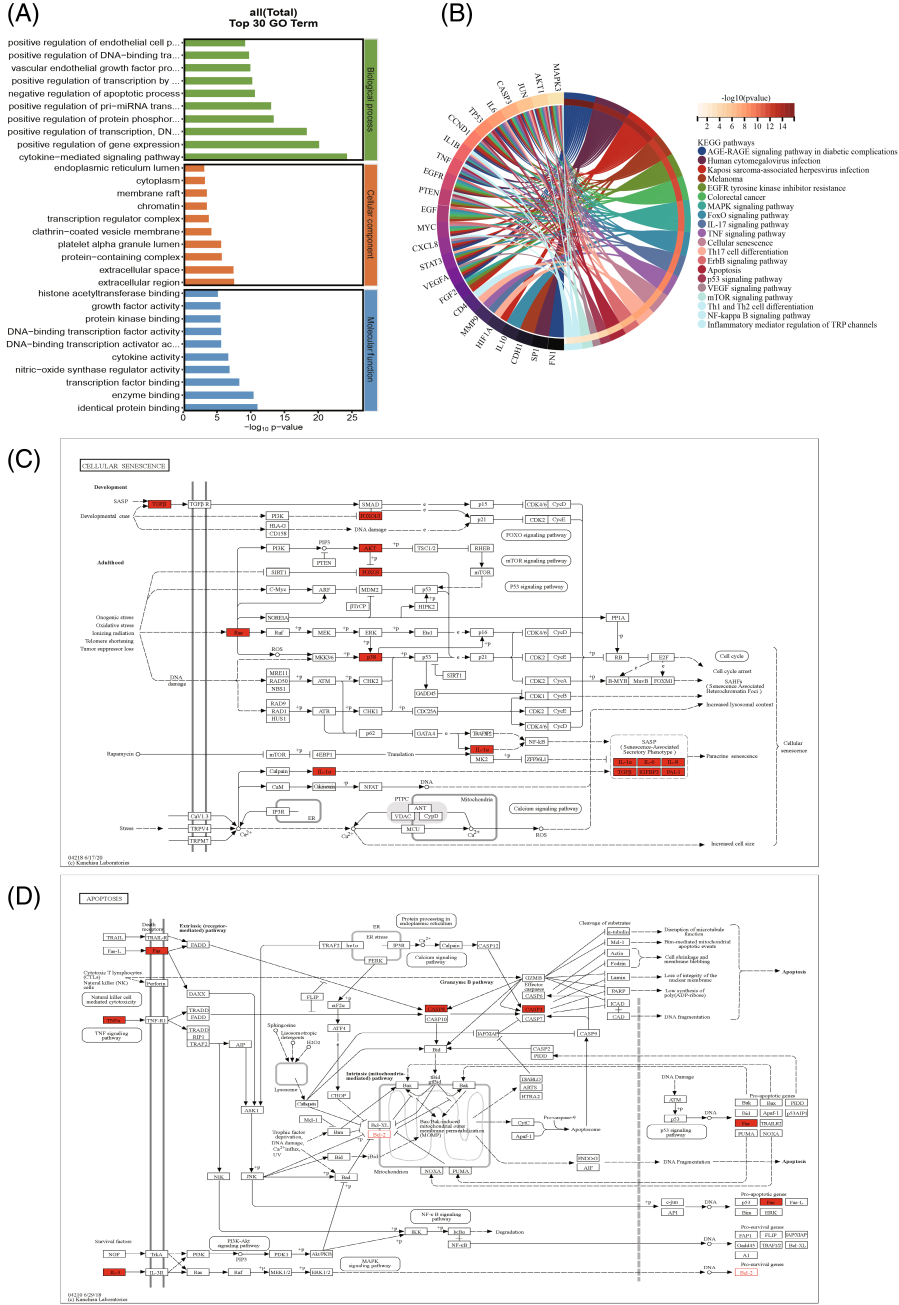
(A) Results of GO enrichment analysis based on hub genes. (B) Results of KEGG enrichment analysis based on hub genes. (C) Cellular senescence signaling pathway and the key expressed genes involved. (D) Apoptosis signaling pathway and the key expressed genes involved (these figures are from the KEGG Signaling Pathway Diagrams website).

We have enriched 27 pivotal genes as a means of exploring the pathogenesis of IVDD disease. GO enrichment analysis revealed a total of 1212 GO entries enriched for hub genes, including 934 entries for biological process (BP), 109 entries for cellular component (CC), and 169 entries for molecular function (MF) (Table S4). The top 10 enrichment results in BP, CC, and MF are presented as bar graphs (Figure 6A), which show that GO analysis during IVDD mainly involves cytokine‐mediated signaling pathway, negative regulation of apoptotic process, cytokine activity, growth factor activity, and membrane raft. KEGG pathway analysis revealed that the IVDD process involves the expression of 136 signaling pathways (Table S5), which were screened to show that these pathways are mainly involved in FoxO signaling pathway, IL‐17 signaling pathway, TNF signaling pathway, CS, p53 signaling pathway, mTOR signaling pathway, Th1 and Th2 cell differentiation, Th17 cell differentiation, and apoptosis (Figure 6B). Among these, the CS and apoptosis signaling pathway (Figure 6C,D) may play a key role in IVDD.

### 3.5 Results of immune infiltration analysis

**FIGURE 7:**
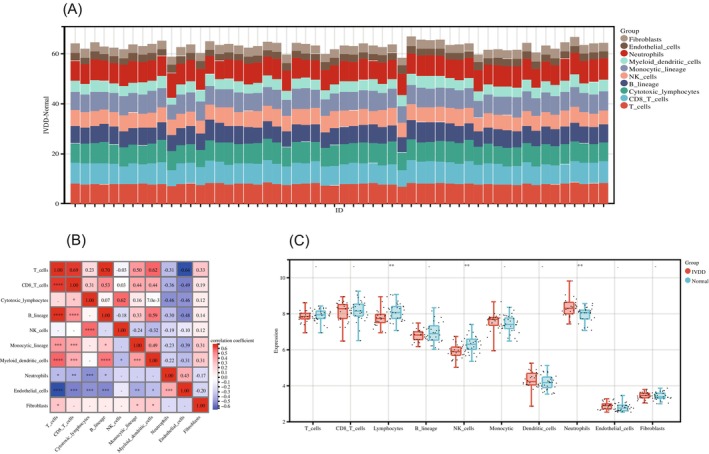
(A) Immune infiltration stacking plot of hub genes. (B) Heat map of immune‐infiltrating cells correlation of hub genes. (C) The statistical significance of the differences between the pivotal gene immune‐infiltrating cell groups is shown.

With the CIBERSORT, algorithm we obtained immuno‐infiltration scores for the hub genes, and the results of the immuno‐infiltration scores are presented in stacked plots (Table S6; Figure 7A). The immune infiltration stack map shows that the main immune cells involved in the hub gene are T cells, NK cells, myeloid dendritic cells, neutrophils, and fibroblasts. Figure 7B shows the correlation between the different immune cells, and Figure 7C shows the statistical significance of these immune cells between the different subgroups. The results show significant differences for NK cells, cytotoxic lymphocytes, and neutrophils (**p < 0.01).

### 3.6 Results of hub gene validation

**FIGURE 8:**
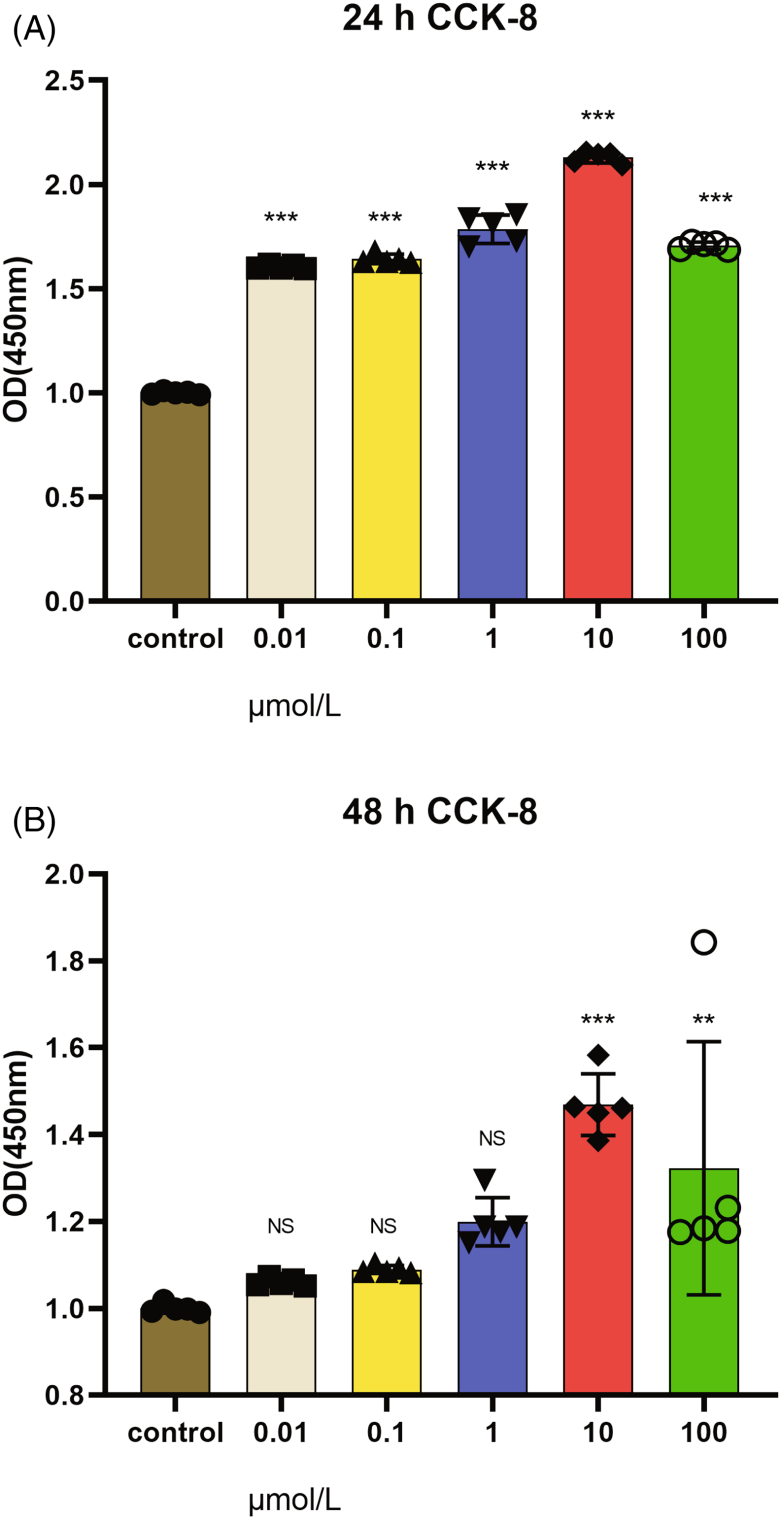
Effect of LPS on the viability of NPCs. (A) NPCs were treated with 0.01, 0.1, 1, 10, and 100 μmol/L LPS for 24 h, and cell proliferation was detected by CCK‐8. *n* = 5, all groups *p < 0.001 versus control. (B) NPCs were treated with 0.01, 0.1, 1, 10, and 100 μmol/L LPS for 48 h, and cell proliferation was detected by CCK‐8. *n* = 5, 0.01, 0.1, 1 μmol/L. These groups were not statistically significant, 10 and 100 μmol/L, *p < 0.01 versus control (comparisons between the two of this group were made using a *t*‐test).

Through bioinformatic data analysis, we clarified that senescence and apoptosis signaling pathways play an important role in the development of IVDD, and based on our group's previous research and literature, we believe that NPCs are important cells in IVDD.^3^, ^14^, ^30^, ^31^ Therefore, we hypothesize that SP1, CASP3, VEGFA, and IL‐6 contribute to the degeneration of NPCs through the regulation of the senescence and apoptosis signaling pathway, which leads to the development of IVDD. First, the effect of LPS on the proliferation of NPCs was investigated. The NPCs were treated with 0.01, 0.1, 1, 10, and 100 μmol/L LPS for 24 and 48 h, respectively, as shown in Figure 8. The proliferative capacity of NPCs was increased after the cells were treated with 0.01, 0.1, 1, and 10 μmol/L LPS for 24 and 48 h, respectively, compared with the control group. The effect was most obvious at 10 μmol/L for 24 h, but proliferation was inhibited when the concentration reached 100 μmol/L (*p* < 0.05), suggesting that LPS can promote the proliferation of NPCs at appropriate concentrations.

**FIGURE 9:**
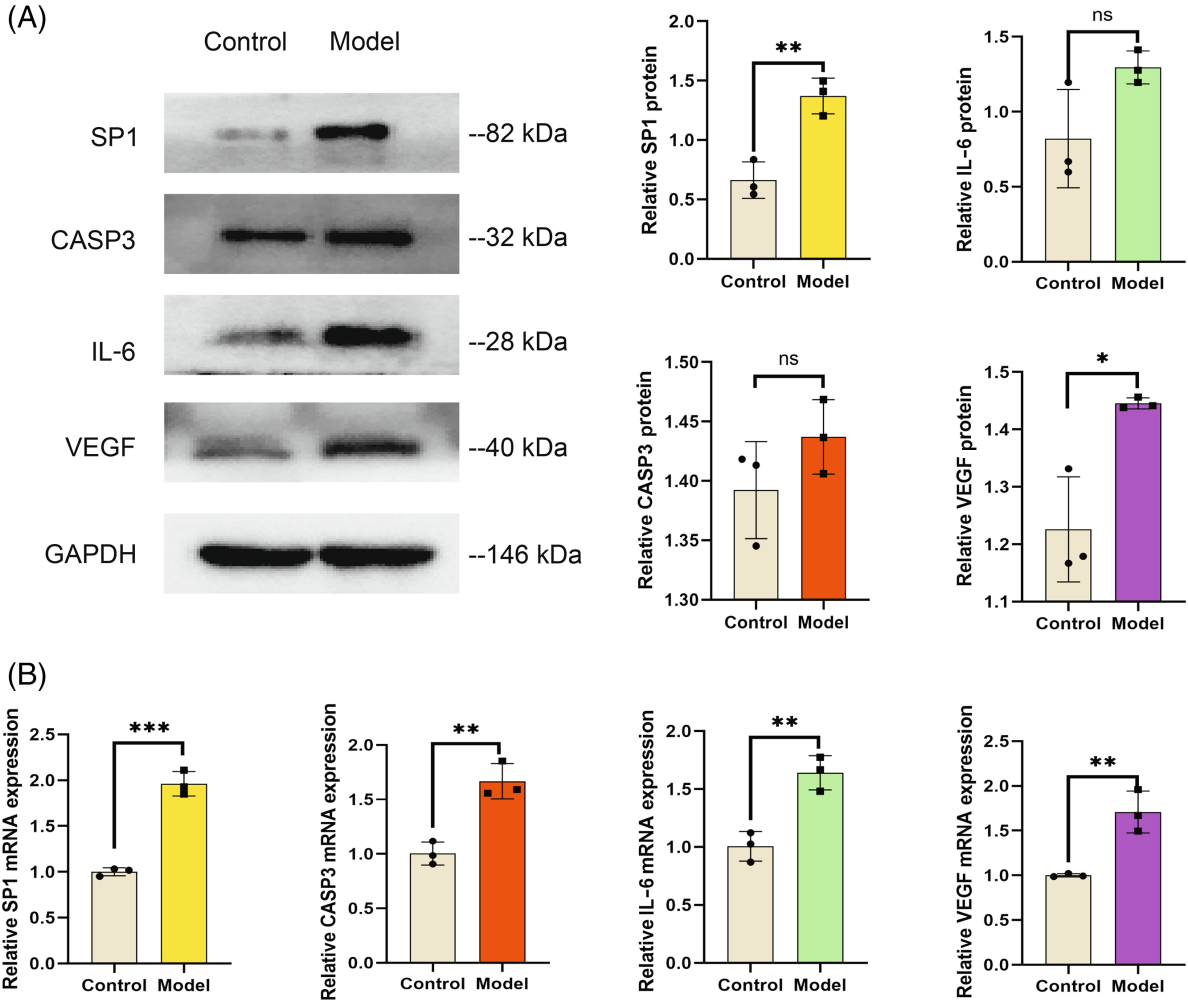
In vitro validation of key pivotal genes. (A) Differences in key pivotal gene proteins between degenerating and normal NPCs. *n* = 3, SP1 *p < 0.01 versus control, IL‐6 NS versus control, CASP3 NS versus control, VEGF *p < 0.05 versus control. (B) Differences in mRNA of key pivotal genes between degenerating and normal NPCs. *n* = 3, SP1 *p < 0.001 versus control, IL‐6 *p < 0.01 versus control, CASP3 *p < 0.01 versus control, VEGF *p < 0.01 versus control (comparisons between the two of this group were made using a *t*‐test).

Based on the experimental results of CCK‐8, we chose 10 μmol/L LPS intervention for 24 h as the grouping criterion for subsequent validation. The cell senescence signaling pathway‐related marker proteins SP1 (**p < 0.01), VEGFA (*p < 0.05), and IL‐6 were highly expressed in the model group by WB assay, and CASP3, a key protein of the apoptotic signaling pathway, was also highly expressed in the model group (Figure 9A). The experimental results of qRT‐PCR also showed high expression of SP1, CASP3, VEGFA, and IL‐6 in the model group (Table S7; Figure 9B). We can see from the WB results that CASP3 is different in the model and control groups in protein levels, but the statistical significance is less significant. However, qPCR showed from the RNA level that CASP3 differed in the model group and the control group, and it was statistically different. Therefore, taken together, CASP3 differed in the model and control groups. The above experimental results provide preliminary evidence that proteins related to senescence and apoptosis pathways are expressed in degenerating NPCs, which would be indicative markers for the development of IVDD.

## 4 DISCUSSION

It is believed that more than 84% of people globally suffer from low back pain, and IVDD is a major pathological element in low back pain, although its pathological mechanism is yet unknown.^32^ The intervertebral disc is a cartilaginous tissue that is located close to the vertebral body and is made up of chondrocyte‐like annulus fibrous (AF) tissue, nucleus pulposus tissue, and cartilaginous endplate (CEP) tissue. IVDD is caused by degeneration and hyalinization of the fibrous ring tissue, water loss in the nucleus pulposus tissue, increased immune cell infiltration, and inflammatory mediators.^33^ The majority of current thinking holds that CS and immunological inflammation are inextricably related to the numerous clinical processes associated with IVDD.^3^, ^14^ Specifically, CS may accelerate disease progression in IVDD by activating immune‐inflammatory responses through ROS, promoting intervertebral disc ECM breakdown, and apoptosis, as well as by inducing oxidative stress and autophagy.^14^ Unfortunately, however, the specific role of aging and immunoinflammatory pathways in IVDD is not yet fully understood.^14^ As a result, we have investigated the mechanism of the function of senescence and immune inflammation in IVDD and provided guidelines for IVDD deep research.

In this study, we initially compared the gene expression of peripheral blood and tissue cells from IVDD patients and healthy controls using the GEO database, and we discovered 27 crucially differentiating genes. Then, using a combination of PPI network, LASSO analysis, and survival analysis, we discovered four crucial genes, SP1, CASP3, VEGFA, and IL‐6, which are linked to CS, immunological inflammation, and apoptosis. Low responsiveness to external signals, such as growth factors and apoptotic stimuli, is a major feature of CS. A significant decrease in senescence‐dependent nucleoplasmic transport‐related genes is responsible for this phenomenon. Specificity protein 1 (Sp1) is the first transcription factor in the nucleoplasmic transport family to be identified and characterized, with a wide range of important biological functions.^34^ It binds to GC‐rich sequences and is involved in regulating the expression of many genes in organisms involved in many aspects of cell life, such as metabolism, cell growth and differentiation, angiogenesis and apoptosis regulation, CS, and so forth.^35^, ^36^ In conclusion, Sp1 may be a key senescence‐associated gene, leading to the formation of a senescence‐dependent functional nuclear barrier and subsequently to low responsiveness to external signals. Increased Sp1 leads to the onset and exacerbation of senescence. Thus, in the model group, Sp1 was increased. In addition, Sp1 was able to reduce the activity of the catabolic factors MMP3, ADAMTS‐4, and SDC4 in NPC, suggesting that Sp1 may be associated with disc‐associated ECM degradation.^37^ The point here is that an increase in SP1 is responsible for the degradation of the ECM.^38^ Levels of proinflammatory cytokines in degenerative discs increase with the progression of IVDD. These cytokines include TNF‐α, IL‐1α, IL‐1β, IL‐6, IL‐17, and various chemokines.^39^ Numerous studies have clarified that IL‐1α, IL‐6, IL‐8, and TGF‐β not only play an important role in the inflammatory response to IVDD, but they are also important components of SASP and play an important role in the induction of CS.^40^ The aspartic protein hydrolases of cysteine are a family of proteases present in the cytoplasm. They are involved in the growth, differentiation, and apoptosis of eukaryotic cells.^41^ It has been demonstrated in recent years to play a role in the pathophysiology of numerous diseases, primarily via controlling apoptosis and inflammatory responses. More and more research are demonstrating that cysteine aspartate is directly linked to the pathophysiology and pathological processes of IVDD, notably in terms of apoptosis and inflammatory responses to IVDD cells. Cysteine 3 is one of them and is crucial to these apoptotic pathways.^41^, ^42^ Members of the cysteinase family induce apoptosis in IVD cells by receiving and transmitting apoptotic signals, leading to an apoptotic cascade response. Thus, indirect or direct regulation of cysteines inhibits apoptosis in intervertebral disc cells and may lead to delayed IVDD and treatment.^43^ Finally, we have experimentally validated the aberrant expression of the senescence hub gene SP1, VEGFA, IL‐6, and the apoptosis key gene CASP3 in degenerating NPCs. Although these four pivotal genes are known to play important roles in various tumors, their roles and mechanisms in disc degeneration remain incompletely understood, and SP1, in particular, has been less well studied. Therefore, we expect that our work will contribute to the direction of future investigations into the function of these four hub genes in IVDD.

The immune system response and inflammation are now widely recognized as important drivers of IVDD pathogenesis and progression.^3^, ^44^, ^45^ We know that under normal physiological conditions, the nucleus pulposus is the largest immune‐privileged tissue in the body, due to the fact that it is covered by the outer layer of the AF and the upper and lower parts of the CEP in a special enclosed space.^46^, ^47^ However, when the degenerating NPCs, it causes a breach in the NPCs‐blood barrier and exposes the NPCs directly to the immune system of the host, ultimately inducing an immune response and infiltration of numerous immune cells, including macrophages, neutrophils, mast cells, T cells (CD4^+^, CD8^+^), Treg cells, and B cells.^11^, ^45^ However, infiltrating immune cells release large amounts of inflammatory factors such as TNF‐α and IL‐1β, which in turn exacerbate the inflammatory response cascade and ECM degradation, contributing to the development of IVDD.^48^, ^49^, ^50^ The CIBERSORT algorithm produced results for immune infiltration based on 27 hub genes. According to further research, IVDD patients had significant immune cell infiltration levels, including activated B cells, CD8^+^ T cells, and natural killer cells. Therefore, we think that these key genes are crucial for the immunological inflammation associated with IVDD. Finally, our study has some limitations and shortcomings. The raw data for the bioinformatics analysis came from mixed cell populations from blood samples and tissue specimens, which made the study lack specimen consistency. And we only used NPCs in the late validation. Therefore, we will unify the specimens or perform multiple validations by collecting blood and tissue specimens in the next study. However, we believe that this study still has little guidance.

## 5 CONCLUSION

In summary, we constructed a model that may be used for disease prediction, based on key differences targets and LASSO technique, forest models, which could predict IVDD illness. Based on 27 hub genes, we discovered that the pathophysiology of IVDD patients is primarily related to aberrant expression of signaling pathways such as CS, apoptosis, and Th17. Finally, we established that four hub genes—SP1, CASP3, VEGFA, and IL‐6—can be used to diagnose IVDD, which is advantageous for its prevention and therapy. In further studies, we will detect the occurrence of CS and apoptosis during IVDD by measuring these four key genes in patients' blood. However, further in‐depth studies on their blood levels and predicted numerical labeling are needed.

## AUTHOR CONTRIBUTIONS

**Chao Song:** Methodology; data analysis; writing—original draft. **Daqian Zhou:** Data analysis; writing—original draft. **Zongchao Liu:** Funding acquisition; providing technical support. **Weiye Cai:** Images analysis. **Fei Liu:** Images analysis. **Yongliang Mei:** Performing the experiments. **Kang Cheng:** Performing the experiments. **Zongchao Liu:** Conceptualization; methodology; supervision; funding acquisition. **Chenyi Huang:** Conceptualization; supervision; writing—review and editing. **Jingwen Chen:** Writing—review and editing. All authors participated in these experiments.

## CONFLICT OF INTEREST STATEMENT

The authors declare no conflict of interest.

## Supporting information


**TABLE S1.** IVDD disease datasets and intersection data based on DisGENET, GeneCards, ALL DEGs.
**TABLE S2.** 162 important gene sets were discovered by the PPI based on intersection data.
**TABLE S3.** 27 hub genes were discovered by the PPI and Cytoscape screenings.
**TABLE S4.** GO enrichment analysis based on 27 hub genes.
**TABLE S5.** KEGG enrichment analysis based on 27 hub genes.
**TABLE S6.** The results of the immuno‐infiltration scores.
**TABLE S7.** The experimental results of qRT‐PCR.

**FIGURE S1.** SP1 Control Model (tree time).
**FIGURE S2.** CASP3 Control Model (tree time).
**FIGURE S3.** IL6 Control Model (tree time).
**FIGURE S4.** VEGF Control Model (tree time).
**FIGURE S5.** GAPDH.

## Data Availability

The original contributions presented in the study are included in the article (Supporting Information Material); further inquiries can be directed to the corresponding authors.
