# Supplementary material for: Bioinformatics‐based discovery of intervertebral disc degeneration biomarkers and immune‐inflammatory infiltrates
Source: JOR Spine. 2023 Dec 22;7(1):e1311. doi: 10.1002/jsp2.1311 (PMC10782055; doi:10.1002/jsp2.1311)

NOTE: All bands from left to right show the different trends in the null-not control and model groups, respectively, and are the result of three replications.

Supplementary Fig 1

SP1 Control Model (tree time)

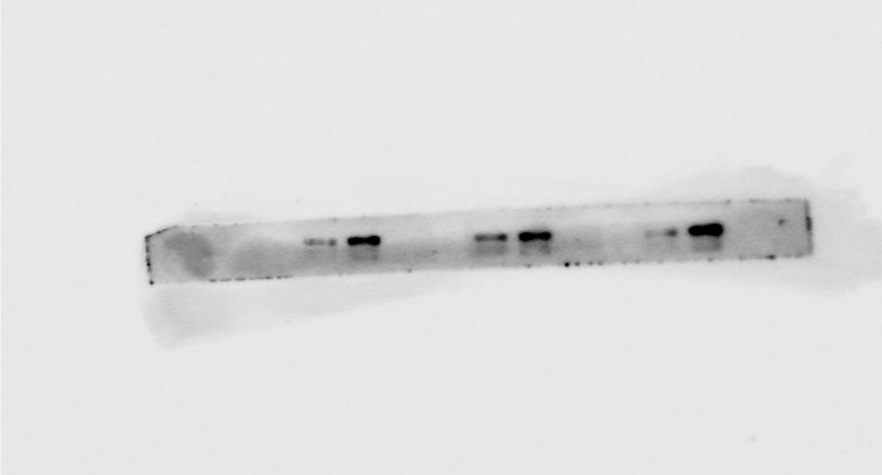

Supplementary Fig 2

CASP3 Control Model (tree time)

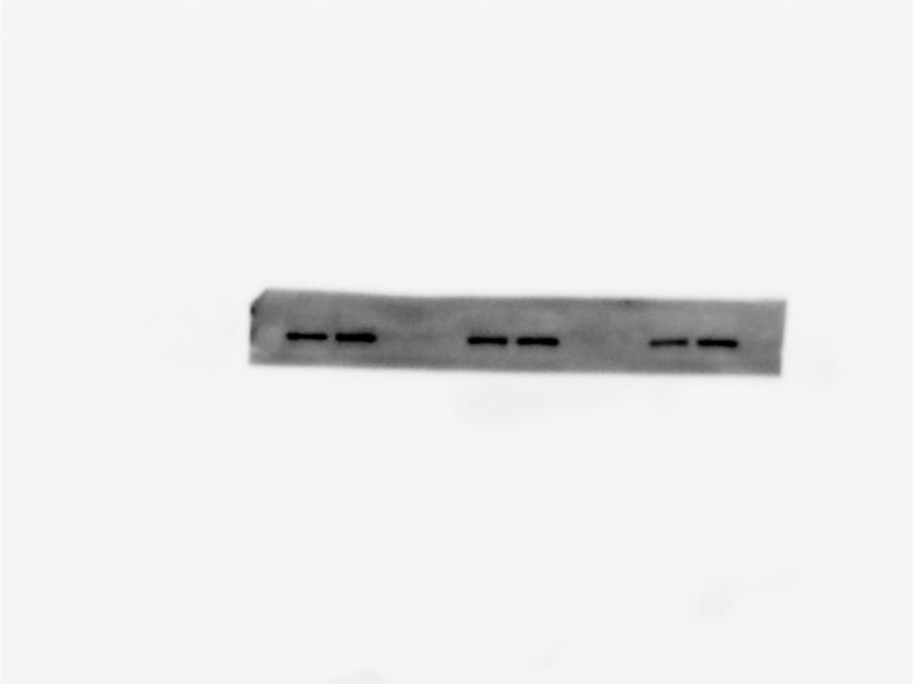

Supplementary Fig 3

IL6 Control Model (tree time)

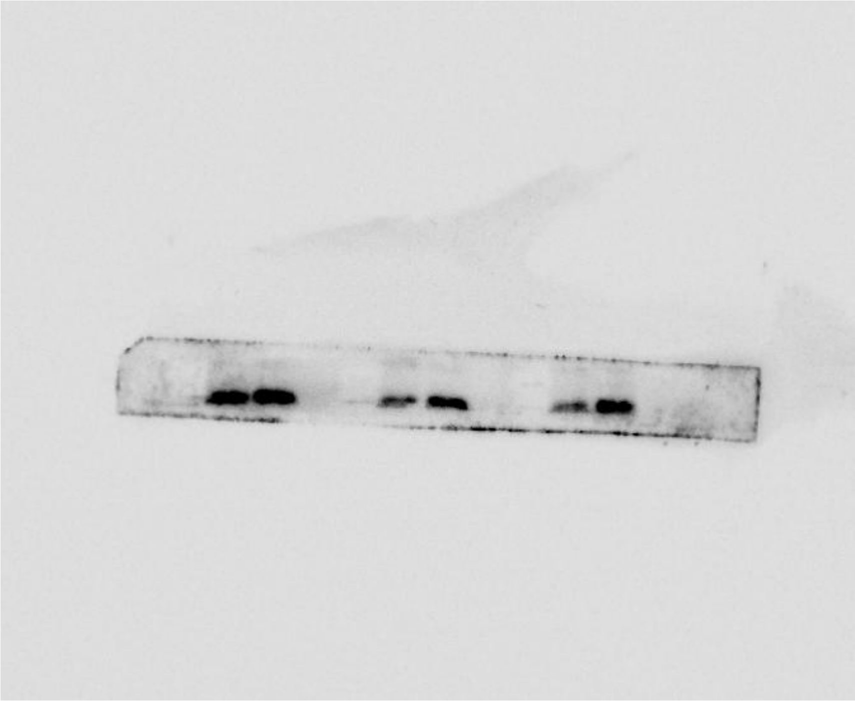

Supplementary Fig 4

VEGF Control Model (tree time)

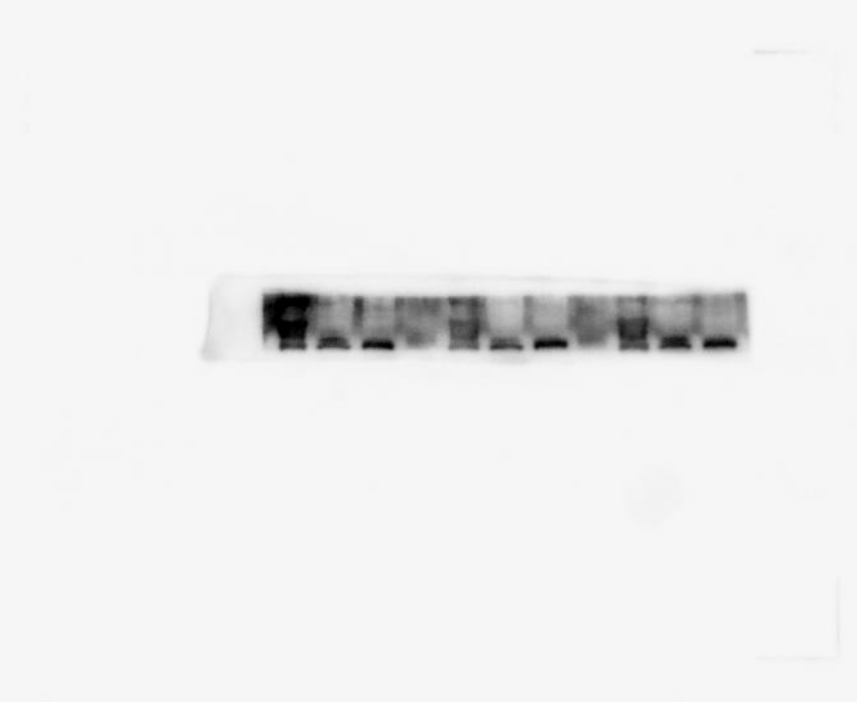

Supplementary Fig 5

GAPDH   sControl   Model (tree time)

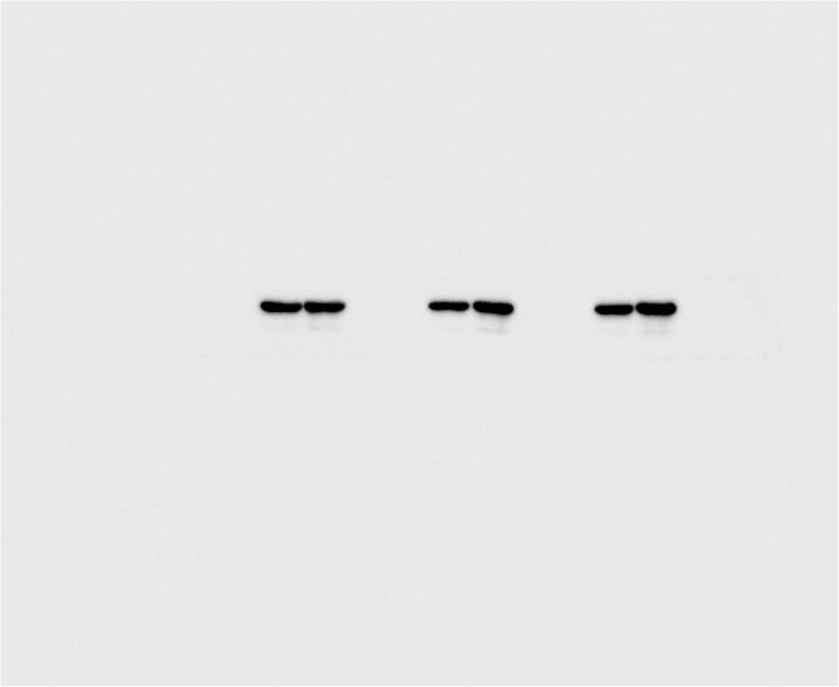

Supplement: Supplementary file 2 — FIGURE S1. SP1 Control Model (tree time). FIGURE S2. CASP3 Control Model (tree time). FIGURE S3. IL6 Control Model (tree time). FIGURE S4. VEGF Control Model (tree time). FIGURE S5. GAPDH. [file JSP2-7-e1311-s001.pdf]
